# Feasibility and Preliminary Effects of a Pressure Sensor–Based Exergame on Cognitive and Sensorimotor Frailty in Low-Income Older Adults Receiving Public Community Care Services: Pilot Randomized Controlled Trial

**DOI:** 10.2196/93467

**Published:** 2026-07-24

**Authors:** Sunmi Song, Suk-Chan Hahm, Seyun Chang, Jinseung Lee, Heewon Kang, Sang Hun Lee, Junesun Kim

**Affiliations:** 1 Rehabilitation Science Program, Department of Health Science Graduate School Korea University Seoul, Seoul Republic of Korea; 2 Transdisciplinary Major in Learning Health Systems, Department of Healthcare Sciences Graduate School Korea University Seoul, Seoul Republic of Korea; 3 Graduate School of Integrative Medicine and Health Cha University Pocheon, Gyeonggi-do Republic of Korea; 4 Geumcheon-gu 205 Gasan digital 1-ro MIDAS Health & Technology Co Seoul, Seoul Republic of Korea; 5 Department of Public Health Administration Daejin University Pocheon, Gyeonggi-do Republic of Korea; 6 Department of Physical Therapy/Health and Environmental Science, Undergraduate School College of Health Science Korea University Seoul, Seoul Republic of Korea

**Keywords:** exergaming, older adults, cognitive function, sensorimotor function, frailty, fall prevention, dual-task training, community-based intervention, digital health, health equity

## Abstract

**Background:**

Age-related cognitive and sensorimotor declines co-occur and reinforce one another, increasing vulnerability to functional dependence and falls. Exergaming offers integrated cognitive-motor training, but most systems lack features that support personalized care for socially vulnerable, community-dwelling older adults. Low-income older adults receiving Public Community Care services bear a disproportionate burden of cognitive and sensorimotor decline, yet remain underrepresented in digital health research. As part of a broader program to develop a digital health monitoring-to-intervention framework, we evaluated a pressure sensor–based exergame designed to deliver targeted cognitive-motor training within the existing Public Community Care infrastructure.

**Objective:**

This study evaluates the feasibility and preliminary effects of the Brain Step exergaming intervention on cognitive and sensorimotor frailty in low-income older adults living alone who were receiving Public Community Care services through a pilot study (study 1) and a randomized controlled trial (RCT; study 2).

**Methods:**

Study 1 (n=15) was a 12-week single-arm pilot study. Study 2 was a parallel-group pilot RCT conducted in Seoul, South Korea. Thirty-one low-income older adults living alone who were receiving Public Community Care services (mean age 81.5 years; 26/31, 84%, female) were randomized to the intervention (n=16) or control (n=15) group. The 16-week intervention used custom hardware with integrated safety bars and arcade-style games that progressed from single- to dual-task challenges. Primary outcomes were cognitive function (Korean Mini-Mental State Examination [K-MMSE]), physical function (Berg Balance Scale [BBS] and Timed Up and Go [TUG]), and sarcopenia indicators (appendicular skeletal muscle mass [ASM], Appendicular Skeletal Muscle Mass Index, and grip strength). Secondary outcomes were the Korean Montreal Cognitive Assessment, physical fitness (Figure-8 Walk, 30-second Sit-to-Stand, and 2-Minute Step Test), and fall incidence. Generalized estimating equations with Benjamini-Hochberg false discovery rate (FDR) correction for multiple testing were applied.

**Results:**

In the single-arm pilot study (study 1), a pre-post increase in BBS scores and a reduction in TUG time survived FDR correction among the primary outcomes (both FDR-adjusted *P*=.002), as did all 3 secondary physical fitness outcomes (Figure-8 Walk, FDR-adjusted *P*=.006; 30-second Sit-to-Stand, FDR-adjusted *P*=.01; and 2-Minute Step Test, FDR-adjusted *P*=.02). The K-MMSE Visuospatial subscale showed an uncorrected pre-post improvement (*P*=.02), with differential risk-stratified patterns for Recall (*P*=.04) and Attention (*P*=.006). In the pilot RCT (study 2), a nominally significant group × time interaction was observed for ASM (*P*=.04, FDR-adjusted *P*=.11), with the intervention group maintaining muscle mass while the control group declined. The risk-stratified analyses showed an uncorrected significant 3-way interaction for K-MMSE Recall (*P*=.01). No findings from study 2 survived FDR correction.

**Conclusions:**

These sequential studies demonstrate the feasibility of delivering a culturally adapted exergaming intervention through the existing Public Community Care infrastructure. The pilot study (study 1) showed FDR-corrected pre-post improvements in balance and mobility within an uncontrolled single-arm design. In the pilot RCT (study 2), no primary outcomes survived FDR correction. The observed preliminary signals for muscle mass preservation and, in exploratory risk-stratified analyses, cognitive improvement in the high-risk subgroups should be regarded as hypothesis-generating only. Adequately powered trials with risk-enriched samples and active comparators are needed to evaluate efficacy.

**Trial Registration:**

ClinicalTrials.gov NCT06664229; https://clinicaltrials.gov/study/NCT06664229

## Introduction

Aging brings concurrent declines across cognitive, sensory, postural, and muscular systems that together threaten independent living in older adults [1,2]. Frailty is increasingly understood not as disability or comorbidity but as a biologic syndrome of decreased physiologic reserve and reduced resistance to stressors, arising from cumulative declines across multiple systems and conferring vulnerability to falls, disability, and loss of independence [3]. This study addresses 2 interrelated aspects of this syndrome. Cognitive frailty denotes the co-occurrence of physical frailty and cognitive impairment in the absence of Alzheimer disease or other dementia—a potentially reversible, pre-neurodegenerative state distinct from physiologic brain aging and, thus, a target for early intervention [4]. Its sensorimotor counterpart, here termed sensorimotor frailty, comprises age-related deficits in proprioception, balance, postural control, and skeletal muscle function that increase the risk of falls and functional dependence [3,5]. It is closely linked to cognitive decline, sarcopenia, and increased fall risk [2,6]. Critically, these 2 aspects are interrelated rather than independent. This interdependence provides the rationale for integrated interventions that simultaneously engage cognitive, sensory, and motor systems rather than targeting each domain in isolation.

The relationship between sensorimotor decline and cognitive aging is bidirectional. Executive subdomains such as attention, sensory integration, and motor planning are strongly associated with postural instability and gait dysfunction [7], while age-related loss of proprioception and sarcopenia independently compromise both mobility and cognitive function [8,9]. Sarcopenia—the progressive loss of skeletal muscle mass and strength with aging—further compounds these vulnerabilities, as lower limb muscle mass is a critical determinant of balance confidence and functional independence in older adults, and sarcopenia is associated with increased risks of falls, fractures, cognitive impairment, and mortality [9-11]. Integrated dual-task approaches that simultaneously engage cognitive and sensorimotor systems may therefore be more effective than targeting either domain alone [12,13]. Such approaches may be particularly relevant for older adults experiencing preclinical functional decline—those who remain independently ambulatory but show individualized deterioration below their own functional baseline. Network meta-analyses have confirmed that exercise interventions significantly reduce both the number of fallers (risk ratio 0.83, 95% CI 0.77-0.89) and the rate of falls (risk ratio 0.79, 95% CI 0.73-0.86), with emerging evidence favoring combined physical-cognitive training [13]. In South Korea, where adults aged 65 years and older constitute 19.2% of the population [14], preserving functional independence through early detection and recovery from these preclinical declines represents a pressing public health priority. While the number of Koreans with dementia is projected to exceed 1 million by 2026 [14,15], up to 45% of dementia cases may be attributable to modifiable risk factors, including sensory loss, physical inactivity, sarcopenia, and depression [16], underscoring the value of interventions that target the functional precursors of cognitive and sensorimotor decline before irreversible impairment occurs.

Exergaming—physically active video gaming that requires whole-body movements—has emerged as a promising approach for delivering integrated cognitive-motor training. Recent randomized controlled trials (RCTs) have demonstrated that exergaming can improve executive function [17,18], dual-task performance [19], and global cognition [18,20] in older adults, with a landmark trial by Sturnieks et al [21] showing that home-based step exergaming and cognitive training reduced fall rates by 26% over 12 months. However, most exergaming research has used commercially available gaming systems—primarily Nintendo Wii, Microsoft Xbox Kinect, or stationary cycling platforms [12,18,19]—which may present significant barriers for older adults with limited technology experience, cognitive impairment, or balance deficits that require postural support during exercise [18].

Despite the growing evidence base for exergaming interventions, critical gaps remain. First, socially vulnerable populations, who bear disproportionate burdens of both falls and cognitive decline, are typically underrepresented in clinical trials, limiting the generalizability of existing evidence [22]. Low-income older adults living alone face multiple barriers to accessing conventional rehabilitation services, including transportation difficulties, financial constraints, and social isolation [23]. Second, most prior RCTs have been conducted in individual home-based settings [12] or laboratory settings [18,19]. While home-based approaches can reduce falls, the social isolation inherent in such formats may limit engagement for some populations. Third, the generational appropriateness of gaming content—identified by Begde et al [12] as a critical determinant of usability and adherence—has not been systematically explored. Most commercially available gaming systems were designed for younger, technology-literate users, and their game interfaces and interaction paradigms may not align with the cultural preferences and prior experiences of today’s older adults.

**Figure 1:**
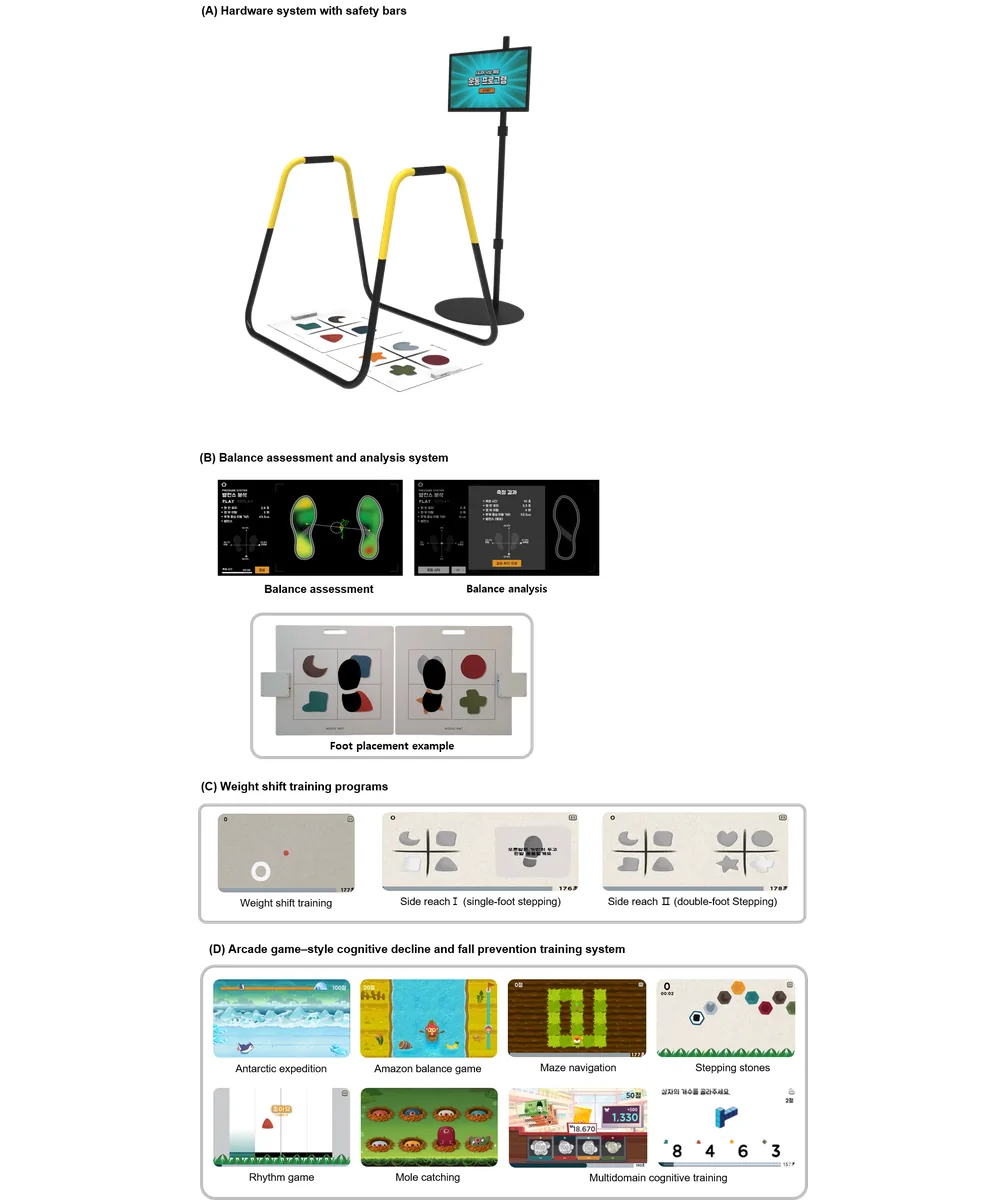
Brain Step system hardware and software components. (A) Hardware system with safety bars. The intervention setup comprises a display screen, dual safety support bars, and a pressure sensor–based response mat with symbol-coded target zones for single- and dual-task training. (B) Balance assessment and analysis system. Real-time visualization of the center of pressure during balance assessment, a postassessment report summarizing balance metrics (duration, sway distance, and weight distribution), and an example of foot placement on the symbol-coded target zones used during training. (C) Weight shift training programs. Exercises targeting lower-limb coordination and weight-shifting control, including Weight Shift Training (directional target tracking), Side Reach I (single-foot stepping), and Side Reach II (double-foot stepping). (D) Arcade game–style cognitive decline and fall prevention training system. Exergames integrating proprioceptive-motor movements with cognitive demands, progressing from motor-focused tasks (Antarctic Expedition, Amazon Balance Game, Maze Navigation, Stepping Stones, Rhythm Game, and Mole Catching) to dual-task cognitive training (multidomain cognitive training targeting orientation, memory, language, visuospatial function, and arithmetic).

To address these gaps, we developed the Brain Step program, a pressure sensor–based exergaming intervention specifically designed for older adults with elevated cognitive and fall risk (Figure 1). The program features custom hardware with integrated safety bars for postural support, culturally adapted arcade-style games familiar to the target generation, and a progressive structure that advances from single-task to cognitive-motor dual-task challenges [12,18]. The intervention was embedded within the existing service infrastructure of a community welfare center, with on-site staff trained as facilitators to ensure sustainability beyond the research period.

This research is part of a 3-phase program to develop and evaluate a digital health monitoring-to-intervention framework for low-income older adults receiving government-supported Community Care services. Phase 1 established a digital health monitoring platform integrating smartphone chatbots, smartwatch sensors, and motion-sensing technology [24], and phase 2 demonstrated that this platform, combined with caregiver-mediated intervention within the public Community Care infrastructure, could maintain functional status in low-income older adults living alone [25]. This study (phase 3) evaluates Brain Step, a pressure sensor–based exergaming intervention designed to deliver targeted cognitive-motor training when signs of functional decline are detected through the monitoring platform.

By embedding the intervention within the existing Public Community Care service infrastructure, this study aims to address a critical translational gap between digital health monitoring and actionable interventions for populations that face structural barriers to conventional rehabilitation services. This research consisted of 2 sequential studies that used a user-centered, community-participatory approach to develop and evaluate the intervention. Study 1 was a single-arm pilot study designed to evaluate the feasibility, safety, and preliminary efficacy of the Brain Step program and to refine the intervention protocol based on participant and staff feedback. Study 2 was a pilot RCT designed to estimate the preliminary effects of the refined 16-week Brain Step intervention on cognitive and sensorimotor frailty in socially vulnerable older adults receiving Public Community Care services. Secondary objectives were to explore the preliminary effects on self-reported health outcomes (depressive symptoms, sleep quality, and pain-related functional limitations) and to examine whether baseline functional risk status moderated intervention effects. Given the exploratory nature of this pilot RCT, we examined whether the intervention group would show preliminary signals of change in cognitive and sensorimotor frailty compared with the control group and whether individuals with higher baseline risk would show differential intervention responses.

## Methods

### Overview

This research consisted of 3 phases conducted at the Seongbuk Municipal Senior Welfare Center (hereafter, the Welfare Center) in Seoul, South Korea. Study 2 participants were recipients of the Korean government’s Public Community Care Service—a nationally standardized, publicly funded program that provides independently ambulatory, low-income older adults living alone with regular caregiver support, including twice-weekly phone calls, weekly home visits, and additional follow-up during episodes of acute illness [26]. Although this study was conducted at a single site, the standardized service protocol—with regulated caregiver work hours (25 hours/week) and caseloads (up to 12 older adults per caregiver)—offers a replicable infrastructure for embedding intervention programs nationwide. A brief prepilot phase tested the initial safety and feasibility of the Brain Step system. Study 1 was a single-arm pilot study designed to evaluate the feasibility and safety of the Brain Step program. Based on the findings of study 1, the intervention protocol was refined and tested in study 2, an RCT. In both studies, participants were predominantly female (38/46, 83%, with 12/15 in the pilot and 26/31 in the RCT), had a mean age of approximately 80 years, and presented with fall risk factors such as a history of falls or gait difficulties. Study 1 recruited community-dwelling older adults from the Welfare Center without income restrictions, whereas study 2 specifically enrolled low-income older adults living alone who were receiving the Public Community Care Service.

### Ethics Approval

This study was conducted in accordance with the Declaration of Helsinki and was approved by the Institutional Review Board of Korea University (approval number KUIRB-2023-0360). The study was registered at ClinicalTrials.gov (NCT06664229). Written informed consent was obtained from all participants before enrollment.

### Pre-Pilot Testing

Before the pilot study, a brief prepilot phase was conducted with 4 older adults to assess the initial safety and feasibility of the Brain Step system. Participants attended sessions 3 times per week for 1 week. This preliminary testing confirmed that the system was safe for use by older adults and that the basic exercises could be completed without adverse events, informing the design of the subsequent pilot study. The overall development approach—progressing from needs assessment through pilot testing to RCT evaluation—was guided by a participatory, user-centered process that engaged older adult participants, community caregivers, and welfare center administrators through qualitative interviews and open-ended feedback collected at posttest. Their input informed device and program design decisions, including the adoption of safety features (integrated support bars), difficulty-level adjustments, and gradual progression from sensorimotor to more complex dual-task training, ensuring that the intervention was contextually appropriate for this population.

### Study 1: Pilot Study

#### Design and Participants

Study 1 was a single-arm pre-post study conducted from March to May 2024. Community-dwelling older adults aged 65 years or older were recruited through bulletin board postings at the community senior welfare center. Inclusion criteria were (1) the ability to communicate and follow instructions and (2) fear of falling. Exclusion criteria were (1) severe sensory impairments (hearing or vision), (2) cognitive or psychiatric impairments (eg, hallucinations), and (3) inability to ambulate independently.

Of 20 older adults screened, 18 met the eligibility criteria and agreed to participate. Three participants withdrew (2 before baseline because of sequelae of back surgery and 1 during the intervention because of scheduling conflicts), resulting in 15 participants (mean age 80.20 years, SD 6.88 years; 12/15, 80%, were women) who completed the study. The characteristics of the pilot study participants are reported in Table S1 in Multimedia Appendix 1.

To examine differential effects according to baseline cognitive function and sensorimotor risk levels, staff at the community senior welfare center assisted with recruitment to ensure balanced enrollment of participants across high- and low-risk categories.

#### Intervention

Participants received the Brain Step program for 12 weeks, attending 50-minute sessions twice weekly. The program consisted of pressure sensor–based exercises, including center of gravity training, single- and double-foot stepping, and exergames (maze navigation, stepping stones, and mole catching). Sessions were conducted in small groups (≤8 participants per session because of space constraints at the Welfare Center) with trained facilitators, including 2 nonclinical welfare center staff members who served as session facilitators from this pilot phase onward, providing guidance and safety monitoring.

#### Outcomes and Analysis

Primary outcomes included cognitive function (Korean Mini-Mental State Examination [K-MMSE]), physical function (Berg Balance Scale [BBS] and Timed Up and Go [TUG]), and sarcopenia-related measures (appendicular skeletal muscle mass [ASM], Appendicular Skeletal Muscle Mass Index [ASMI], and handgrip strength).

Secondary outcomes included physical fitness measures (30-second Sit-to-Stand, 2-Minute Step Test, and Figure-8 Walk) and device and program usability. Participants also completed qualitative interviews and satisfaction surveys. Pre-post changes were analyzed using generalized estimating equations (GEEs). Participant feedback was collected through focus group interviews with participants and senior welfare center staff.

### Study 2: RCT

#### Design

Study 2 was a parallel-group RCT. Recruitment began in August 2024, the intervention was delivered from November 2024 to March 2025, and the final assessment was completed in April 2025. Participants were assessed at 3 time points: baseline (preintervention), 8 weeks (mid-intervention), and 16 weeks (postintervention).

#### Participants

Study 2 targeted low-income older adults living alone who were receiving the Customized Community Care Service described above. Participants were identified through the Community Care service database at the Welfare Center. Community Care providers facilitated recruitment by identifying eligible older adults from their caseloads, arranging initial contact with the research team, and coordinating transportation to the Welfare Center for intervention sessions. Through this involvement, Community Care providers also learned the intervention procedures and progressively assumed facilitator roles, further supporting the program’s sustainability within routine welfare center operations. The inclusion and exclusion criteria were identical to those of study 1.

#### Sample Size

Sample size was calculated using G*Power version 3.1.9.7. Based on a repeated-measures analysis of variance with a within-between interaction, a medium effect size (Cohen *f*=0.25) [27] was assumed. This assumption was based on meta-analytic evidence from exergaming interventions reporting pooled effect sizes of standardized mean difference of 0.35 for executive function [28] and standardized mean difference of 0.36-0.78 for balance outcomes in older adults [29], consistent with prior meta-analytic evidence [17]. Assuming an α level of .05, statistical power of 0.80, 2 groups, and 3 measurement time points, a minimum of 28 participants was required. Accounting for a 15% (5/33) dropout rate, the target sample size was 33 participants. To ensure balance between groups, 34 participants were planned for recruitment.

#### Randomization and Blinding

The random allocation sequence was generated using block randomization with a fixed block size of 4 by an independent researcher who was not involved in participant recruitment, intervention delivery, or outcome assessment. The allocation sequence was concealed from the other investigators until the time of assignment. Owing to the nature of the intervention, participants were not blinded and were aware of their group assignment, whereas outcome assessors were blinded to group allocation to minimize detection bias.

#### Intervention

As shown in Figure 1, the Brain Step program consists of a large pressure sensor mat (1100 mm × 1100 mm) with 4 quadrants and 8 response zones, a display screen, and integrated safety bars.

Participants attended 50-minute sessions twice weekly for 16 weeks (32 sessions total). Sessions were conducted in small groups of up to 8 participants (limited by the available space and time at the Welfare Center), with morning and afternoon sessions available. Each session was led by 1 trained facilitator, with 1-2 research assistants providing individual guidance and safety monitoring. Instructional videos were provided to explain the program objectives and demonstrate each game task. Facilitators received training in device operation, safety monitoring, emergency procedures, and participant guidance before program initiation. To prepare for technology-assisted translation into routine community care practice, 2 welfare center staff members—who were not specialists in cognitive or physical interventions—were intentionally included as facilitator trainees, reflecting the typical workforce of community-based older adult care settings. Their operational needs were elicited through qualitative interviews and incorporated into the program design, including adjustable difficulty levels, automated game modules, and instructional videos demonstrating each task to support independent program delivery. Intervention fidelity was monitored through session checklists completed by research assistants and weekly supervision meetings. Adherence was tracked through individual session attendance records and game completion rates documented automatically by the Brain Step system.

As shown in Table 1, the main trial program was structured into 3 progressive phases. Phase 1 (weeks 1-4) focused on single-task training, including weight-shift exercises with real-time center of pressure feedback and side-reaching exercises (single- and double-foot stepping) to strengthen ankle mobility and lower limb muscle function. Phase 2 (weeks 5 and 6) introduced measurement system level 2, combining physical exercises with location memory tasks and learning system games to increase task complexity. Phase 3 (weeks 7-16) emphasized dual-task training through multidomain cognitive training targeting image association, arithmetic, and recognition. Participants selected answers by stepping on designated target zones among the 8 positions on the pressure sensor mat, thereby integrating cognitive decision-making with weight-shifting movements.

The software comprised 3 components (Figure 1): a balance assessment system (Figure 1B) providing real-time center-of-pressure visualization, a weight-shift training system (Figure 1C), and a cognitive-motor training system (Figure 1D) featuring 7 exergames inspired by classic arcade games from the 1980s and 1990s to enhance engagement through nostalgic familiarity. Detailed descriptions of each game are provided in the Methods S1 in Multimedia Appendix 1.

**Table 1:** Table1. Brain step program structure and progression.

Phase	Weeks	Training focus	Tasks	Task type
1	1-4	Ankle mobility and lower limb strengthening	Center of gravity training and side reach (single/double foot stepping)	Single task
2	5 and 6	Memory and spatial cognition	Maze navigation, stepping stones, and mole catching	Increased complexity
3	7-16	Cognitive-motor integration	Cognitive training quizzes (image association, arithmetic, and recognition) with proprioceptive-motor movements	Dual task

#### Control Group

Participants in the control group received no intervention during the 16-week study period and attended only the 3 assessment sessions (baseline, 8 weeks, and 16 weeks). A passive (no-contact) control was selected at this feasibility stage to establish whether the intervention could be delivered and whether participants would engage with it in the real-world Public Community Care setting. As the intervention was delivered on-site within the existing infrastructure of the community welfare center, an active or attention-matched comparator was not logistically feasible at this stage. Limited space availability and a shortage of on-site staff precluded implementation of a parallel active-control program. Our priority was to evaluate the real-world applicability of the intervention.

#### Risk Stratification

Participants were stratified into high-risk (n=17) and low-risk (n=14) groups based on initial screening assessments. High-risk classification required the presence of 2 or more of the following: a history of recurrent falls, gait abnormalities, or cognitive test scores indicating mild cognitive impairment (K-MMSE ≤21 and Montreal Cognitive Assessment [MoCA] <23). This stratification was prespecified during the study design phase. Community stakeholders requested the inclusion of participants at the highest cognitive and sensorimotor risk, and the pilot study findings of differential response patterns informed the decision to classify participants by risk level using pretest screening (K-MMSE and fall history).

#### Outcome Measures

Comprehensive health outcomes were assessed to provide individualized feedback within the digital-human integrated personalized care framework. Outcomes were organized into 3 tiers: primary outcomes for which intervention effects were hypothesized, secondary outcomes to capture more detailed functional changes, and self-reported health outcomes routinely monitored in Community Care services. Primary outcomes included cognitive function (K-MMSE total and subscale scores), physical function (BBS and TUG), and sarcopenia indicators (ASM, ASMI, and grip strength). The K-MMSE [30] evaluates orientation, recall, attention/calculation, language, and visuospatial function (total score 0-30). Balance was measured using the Korean version of the BBS (14 items; total score 0-56) [31,32]. The TUG measured the time (in seconds) required to rise from a chair, walk 3 m, turn, return, and sit down [33]. Sarcopenia-related measures were assessed according to the Asian Working Group for Sarcopenia 2019 criteria [34]. ASM was measured using bioelectrical impedance analysis (InBody 370), and ASMI was calculated as ASM/height^2^ (kg/m^2^). Grip strength was measured using a dynamometer (CAMRY EH101) [35,36], with the highest value from 3 trials of the dominant hand used for analysis.

Secondary outcomes were complementary, hypothesis-generating measures, including cognitive function (Korean Montreal Cognitive Assessment [MoCA-K] total and subscale scores), physical fitness (Figure-8 Walk Test, 30-second Sit-to-Stand, and 2-Minute Step Test [36,37]), and fall incidence. The MoCA-K [38,39] was included as a secondary cognitive measure because the K-MMSE has known ceiling effects in detecting subtle cognitive changes in individuals with mild cognitive impairment [40]. The MoCA-K provides greater sensitivity for mild cognitive impairment through more demanding assessments of executive function, visuospatial ability, and attention (total score 0-30) [39].

Fall incidence was assessed at 3 time points. At baseline, participants completed a screening question (Have you experienced a fall in the past year?). At the immediate postintervention assessment, they were asked, “Have you experienced a fall during the past 16 weeks?” At the 1-year postintervention follow-up, fall incidence was determined from community caregiver fall incident reports shared by the municipal welfare center as part of routine Public Community Care service monitoring. Fall incidence was included as a secondary outcome to complement the primary balance and mobility measures.

Other outcomes included sleep quality (Pittsburgh Sleep Quality Index [PSQI]; total score 0-21, with higher scores indicating poorer sleep quality) [41], depressive symptoms (Geriatric Depression Scale-Short Form [GDS-SF]) [42], and pain-related functional limitations (Western Ontario and McMaster Universities Osteoarthritis Index [WOMAC]) [43].

#### Game Performance Metrics

Game performance data were automatically recorded throughout the 16-week intervention for all participants in the intervention group (n=16). A total of 63 performance indicators were generated across 9 games, including play count, average score, play time, reaction time, and game-specific metrics.

#### Statistical Analysis

Baseline characteristics were compared between groups to assess randomization balance using *t* tests (unpaired and 2-tailed) for continuous variables and chi-square or Fisher exact tests for categorical variables. The primary analysis examined intervention effects on primary, secondary, and other health outcomes using GEEs to account for the correlation among repeated measures within participants. GEE models specified an exchangeable working correlation structure with robust SEs. All randomized participants were analyzed according to their assigned groups (intention-to-treat). Missing observations were handled at the observation level using available-case analysis without imputation, with all 31 randomized participants retained in their originally assigned groups and each contributing all available time points to the estimation [44-46]. For grip strength, 1 participant could not be assessed because of hand surgery unrelated to the intervention and was therefore excluded from the grip strength model (n=30). All other outcomes included all 31 randomized participants (see the “Data Exclusion and Missing Data” section).

The full model included group (intervention vs control), time (baseline, 8 weeks, and 16 weeks), and the group × time interaction to examine whether intervention effects were significant. Risk-stratified analyses were also conducted, as prespecified during the study design phase. Years of education were included as a covariate in the models for cognitive function to account for potential confounding. Given the number of outcome variables tested, the Benjamini-Hochberg false discovery rate (FDR) correction was applied within the 6 primary outcomes (K-MMSE total, BBS, TUG, ASM, ASMI, and grip strength; 6 comparisons) at *q*=0.05. Secondary outcomes (MoCA-K total, Figure-8 Walk, 30-second Sit-to-Stand, 2-Minute Step Test, and fall incidence) and other outcomes (PSQI, GDS-SF, and WOMAC) were reported with unadjusted *P* values only. Subscale scores were reported descriptively without FDR correction. These analyses were distinguished by both outcome tier and inferential status. Confirmatory inference was restricted to the 6 prespecified primary outcomes with FDR correction. The secondary and other outcomes, the K-MMSE subscale analyses, and the prespecified risk-stratified subgroup analyses were exploratory and hypothesis-generating and were reported with unadjusted *P* values without confirmatory interpretation. The only post hoc, data-driven analyses were the exploratory correlations between game performance metrics and clinical outcomes (Figure S1 in Multimedia Appendix 1).

An exploratory analysis examined the associations between game performance metrics and significant changes in outcome measures in study 2, with *P*<.05 applied as the exploratory threshold for identifying significant outcome changes in the intervention group. Pearson correlation coefficients were calculated between game performance metrics and nominally significant changes in primary outcomes (pre-to-post and mid-to-post; for ASM, pre-to-post change, *P*=.04). For K-MMSE Recall (*P*=.01) and ASMI (*P*=.05), analyses were conducted in the high-risk subgroup (n=8) based on significant group × risk × time interactions. For ASM, analyses were conducted in the full intervention group (n=14).

Statistical significance was set at *P*<.05 (2-tailed). The FDR correction described above was applied to account for multiple outcome comparisons. The study was not powered to detect intervention effects for all outcomes or subgroup effects. All risk-stratified and correlation analyses were exploratory and hypothesis-generating. Effect sizes were calculated as Cohen *d* based on the between-group differences in pre-to-post change scores divided by the pooled SD of the change scores, with 95% CIs [27]. Analyses were performed using Python 3.12 (Python Foundation) with the statsmodels package for GEE models and SciPy for correlation analyses. Additional statistical details, including the analysis of fall incidence outcomes, are provided in Methods S1 in Multimedia Appendix 1.

#### Data Exclusion and Missing Data

Of the 31 randomized participants, all completed the baseline assessment except for 1 intervention participant who declined the grip strength measurement because of a history of bilateral hand surgery. This participant subsequently withdrew before the 8-week assessment. By the 8-week midpoint, 5 participants had withdrawn (2 intervention and 3 control), leaving 26 participants with data at that time point. By the 16-week postintervention assessment, 1 additional control participant had withdrawn because of scheduling conflicts, leaving 25 participants with data at that time point. The full longitudinal data set thus comprised 82 participant-by-time point observations across the 3 assessments.

Missing data resulted primarily from participant attrition rather than item-level nonresponse and were not imputed. All GEE analyses used available-case analysis at the observation level. Each participant-by-time point record was included when the outcome value was nonmissing, whereas records missing key covariates (group assignment or baseline risk stratum) were excluded. This approach leverages GEE’s ability to accommodate unbalanced repeated-measures data without listwise deletion at the participant level [45,46], such that all 31 randomized participants were retained in their originally assigned groups, with each contributing all available observations to the estimation.

## Results

### Study 1: Pilot Study Results

The pilot study results are presented in Table 2. Among the primary outcomes, significant pre-post changes that survived FDR correction were observed for the BBS (*P*<.001, FDR-adjusted *P*=.002) and TUG (*P*<.001, FDR-adjusted *P*=.002). ASM (*P*=.04) and ASMI (*P*=.047) showed modest decreases but did not survive FDR correction (FDR-adjusted *P*=.07). The K-MMSE Visuospatial subscale showed a significant uncorrected pre-post change (*P*=.02). Among the secondary outcomes (Table S2 in Multimedia Appendix 1), all 3 measures survived FDR correction: Figure-8 Walk (*P*=.002, FDR-adjusted *P*=.006), 30-second Sit-to-Stand (*P*=.007, FDR-adjusted *P*=.01), and 2-Minute Step Test (*P*=.02, FDR-adjusted *P*=.02).

**Figure 2:**
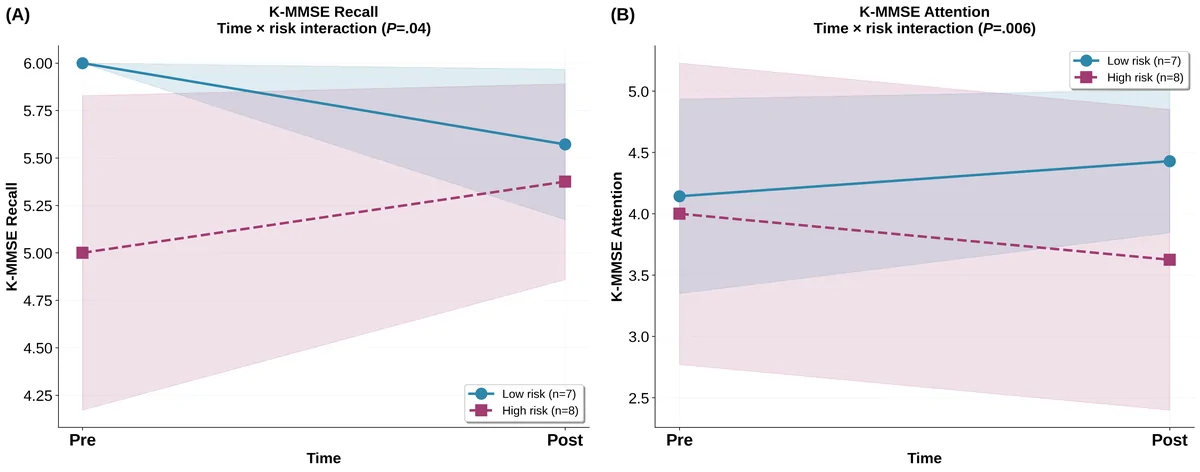
Risk-stratified effects on K-MMSE subscale scores in study 1 (n=15). (A) Memory: significant time × risk interaction (*P*=.04), with improvement observed only in the high-risk group. (B) Attention: significant time × risk interaction (*P*=.006), with improvement observed only in the low-risk group. Shaded areas indicate 95% CIs around the estimated means. K-MMSE: Korean Mini-Mental State Examination.

Risk-stratified analyses revealed nominally significant time × risk interactions for the K-MMSE Memory (*P*=.04) and Attention (*P*=.006) subscales, with differential patterns according to risk group (Figure 2).

Based on the pilot findings and participant feedback, the intervention protocol was refined for study 2. Instructional videos were added, real-time feedback was enhanced, the program duration was extended from 12 to 16 weeks, and a midpoint assessment was added at 8 weeks.

**Table 2:** Comparison of cognitive and sensorimotor frailty over time for pilot testing.^a^

Variable				Intervention group (n=15)				*P* value		FDR^b^ *P* value
				Pre		Post				
**Cognitive frailty, mean (SD)**										
	**Korean Mini-Mental State Examination, total ^c^**			24.47 (3.23)		24.53 (2.85)		.83		.83
		Orientation	9.47 (0.99)		9.40 (1.12)		.74		N/A^d^	
		Recall	5.47 (0.99)		5.47 (0.64)		>.99		N/A	
		Attention	4.07 (1.44)		4.00 (1.41)		.65		N/A	
		Language	7.87 (0.35)		7.80 (0.41)		.30		N/A	
		Visuospatial	0.60 (0.51)		0.87 (0.35)		.02^e^		N/A	
**Sensorimotor frailty, mean (SD)**										
	Berg Balance Scale (score)			51.10 (3.92)		53.20 (2.70)		<.001^e^		.002^e^
	Timed Up and Go (seconds)			8.45 (2.47)		7.41 (2.27)		<.001^e^		.002^e^
	Appendicular skeletal muscle mass (kg)			14.65 (2.18)		14.31 (2.44)		.04^e^		.07
	Appendicular Skeletal Muscle Mass Index (kg/m^2^)			6.14 (0.74)		5.99 (0.74)		.047^e^		.07
Grip strength (kg)				22.01 (4.58)		22.95 (5.36)		.09		.11

^a^Pre-post changes were analyzed using generalized estimating equations with an exchangeable correlation structure. The Benjamini-Hochberg FDR correction (*q*=0.05) was applied within the 6 primary outcomes.

^b^FDR: false discovery rate.

^c^Analyses with Korean Mini-Mental State Examination outcomes were adjusted for years of education.

^d^N/A: not applicable.

^e^*P*<.05.

### Study 2: The Main RCT Results

#### Participant Characteristics

**Figure 3:**
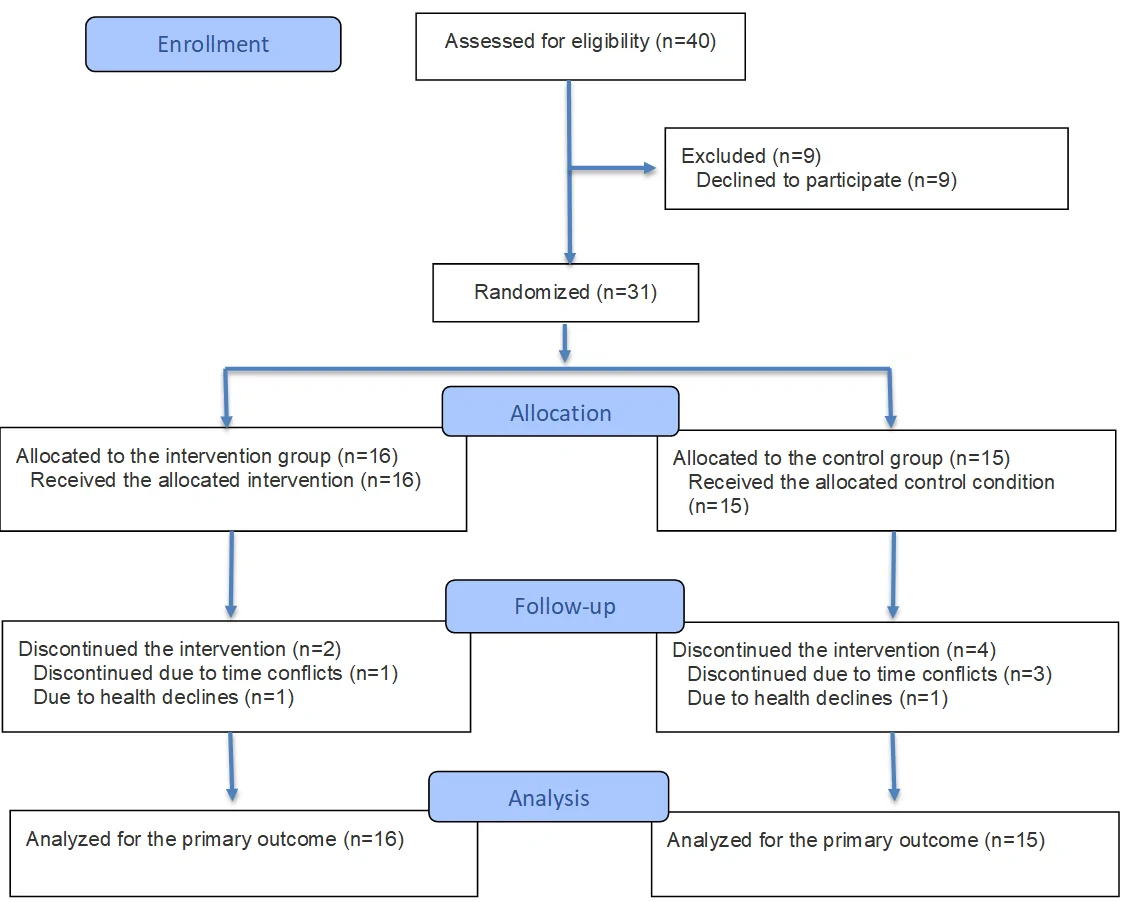
CONSORT (Consolidated Standards of Reporting Trials) flowchart.

Participant flow is presented in Figure 3. Of the 40 older adults screened, 31 were randomized to the intervention (n=16) or control (n=15) group. Six participants withdrew during the study. All 31 randomized participants were included in the intention-to-treat analysis (82 total observations across 3 time points). See Multimedia Appendix 2 for the study’s CONSORT-EHEALTH (Consolidated Standards of Reporting Trials of Electronic and Mobile Health Applications and Online Telehealth) checklist.

Baseline characteristics are presented in Table 3. Participants (mean age 81.5 years; 26/31, 84%, female) were low-income older adults living alone who were receiving Public Community Care services. The groups were comparable at baseline (see Table 3 for *P* values).

**Table 3:** Baseline characteristics of older adult participants by groups for the randomized controlled trial.

Variable			Values					*P* values	
			Intervention (n=16)		Control (n=15)				
**Demographic variables**									
	Age (years), mean (SD)	82.06 (5.42)		80.93 (4.92)		.55			
	Sex (women), n (%)	13 (81)		13 (87)		>.99			
	Years of education, mean (SD)	5.88 (4.86)		4.87 (3.23)		.86			
	Working (yes), n (%)	7 (44)		8 (53)		.86			
**Monthly income (KRW^a^), n (%)**									.23
	Less than 500,000	5 (31)		2 (13)					
	500,001-1,000,000	10 (63)		11 (73)					
	1,000,000 or more	1 (6)		2 (13)					
**Charlson Comorbidity Index, n (%)**									.47
	0	9 (56)		8 (53)					
	1-2	7 (44)		5 (33)					
	3 or more	0 (0)		2 (13)					
**Cognitive functions, mean (SD)**									
	Montreal Cognitive Assessment	18.81 (4.18)		18.93 (4.08)		.56			
	Korean Mini-Mental State Examination	24.44 (2.90)		25.73 (1.87)		.28			
**Sensorimotor function and body composition, mean (SD)**									
	BMI (kg/m^2^)	26.54 (4.01)		24.55 (3.76)		.16			
	Berg Balance Scale	52.88 (1.76)		52.73 (2.46)		.92			
	Appendicular skeletal muscle (kg)	15.35 (4.48)		17.03 (6.00)		.28			
	Appendicular Skeletal Muscle Index (kg/m^2^)	6.41 (1.22)		6.96 (2.40)		.71			
	Grip strength	21.16 (6.88)		20.79 (4.61)		.86			
**Fall incidence at baseline, n (%)**									
	Falls in the past year (yes)	5 (31)		6 (40)		.61			
**Other health outcomes, mean (SD)**									
	Pittsburg Sleep Quality Index	8.12 (3.90)		9.47 (4.85)		.40			
	Geriatric Depression Scale-Short Form	3.56 (3.93)		3.33 (2.92)		.86			
	Western Ontario and McMaster Universities Osteoarthritis Index total	40.81 (15.36)		48.93 (17.73)		.22			

^a^US $1=1344 Korean won (KRW).

#### Intervention Effects on Primary Outcomes

As shown in Table 4, there were no significant group × time interactions for cognitive function. The K-MMSE total score showed no significant interaction (*P*=.27, FDR-adjusted *P*=.38). No significant interactions were observed for the K-MMSE subscales (Orientation, *P*=.36; Recall, *P*=.09; Attention, *P*=.37; Language, *P*=.27; and Visuospatial, *P*=.12).

A nominally significant group × time interaction was observed for the sarcopenia-related index ASM (*P*=.04; Cohen *d*=0.39, 95% CI −0.41 to 1.18). The intervention group maintained ASM over the 16-week period (baseline: 15.35 kg; postintervention: 15.77 kg), whereas the control group showed a decline (baseline: 17.03 kg; postintervention: 15.48 kg; Table 4). The group × time interaction for ASMI did not reach statistical significance (*P*=.05; *d*=0.38, 95% CI −0.41 to 1.18). This pattern is consistent with possible preservation of muscle mass, although the finding did not survive FDR correction. The BBS showed a marginally significant group × time interaction (*P*=.05; *d*=−0.27), likely reflecting a ceiling effect given the high baseline scores in both groups (intervention: 52.88; control: 52.73 of a maximum score of 56). No significant group × time interactions were observed for the other sensorimotor frailty measures (Timed Up and Go, *P*=.65; for grip strength, *P*=.32).

After FDR correction, none of the group × time interactions remained statistically significant (all FDR-adjusted *P*>.16; see Table 4). However, effect sizes are presented to provide context for the observed differences. Before correction, the nominally significant findings showed small-to-medium effect sizes (ASM *d*=0.39; ASMI *d*=0.38), and the risk-stratified analyses revealed medium-to-large effect sizes among high-risk participants for K-MMSE Recall (*d*=0.73) and ASMI (*d*=0.90), warranting confirmation in larger trials with risk-enriched samples.

**Table 4:** Comparison of cognitive and sensorimotor frailty outcomes between intervention and control groups over time (n=31).

Variable			Intervention (n=16)			Control (n=15)				Group × time^a^					*d* ^b^
			Pre, mean (SD)	Post, mean (SD)	Pre, mean (SD)		Post, mean (SD)			*P* value		FDR^c,d^ *P* value			
**Cognitive frailty**															
	**Korean Mini-Mental State Examination^e^, total**		24.44 (2.90)	22.00 (3.01)	25.73 (1.87)		23.27 (2.24)			.27		.38		–0.17	
		Orientation	9.12 (0.89)	9.36 (1.08)	9.13 (1.30)		9.73 (0.47)			.36		N/A^f^		–0.63	
		Recall	4.88 (1.09)	4.86 (0.95)	5.00 (0.93)		5.00 (1.10)			.09		N/A		–0.29	
		Attention	2.38 (1.75)	2.64 (1.69)	3.13 (1.36)		3.00 (1.55)			.37		N/A		0.30	
		Language	7.56 (0.51)	7.43 (0.76)	7.60 (0.63)		7.73 (0.47)			.27		N/A		–0.08	
		Visuospatial	0.50 (0.52)	0.71 (0.47)	0.87 (0.35)		0.82 (0.40)			.12		N/A		0.69	
**Sensorimotor frailty**															
	Berg Balance Scale		52.88 (1.76)	53.82 (1.15)	52.73 (2.46)			53.82 (2.21)	.05		.11		–0.27		
	Timed Up and Go (seconds)		7.67 (1.80)	6.86 (0.82)	7.97 (1.99)			7.54 (1.34)	.65		.65		–0.03		
	Appendicular skeletal muscle mass (kg)		15.35 (4.48)	15.77 (4.56)	17.03 (6.00)			15.48 (3.06)	.04^g^		.11		0.39		
	Appendicular Skeletal Muscle Mass Index (kg/m^2^)		6.41 (1.22)	6.56 (1.16)	6.96 (2.40)			6.36 (1.04)	.05		.11		0.38		
	Grip strength (kg)		21.16 (6.88)	23.27 (5.37)	20.79 (4.61)			19.71 (3.73)	.32		.38		0.50		

^a^Group × time interaction effects were analyzed using generalized estimating equations with exchangeable correlation structure.

^b^Cohen *d* was calculated from between-group differences in change scores divided by pooled SD.

^c^FDR: false discovery rate.

^d^The Benjamini-Hochberg FDR correction (*q*=0.05) was applied within the 6 primary outcomes (Korean Mini-Mental State Examination total, Berg Balance Scale, Timed Up and Go, appendicular skeletal muscle mass, Appendicular Skeletal Muscle Mass Index, and grip strength). No outcomes reached significance after FDR correction.

^e^The Korean Mini-Mental State Examination models were adjusted for years of education.

^f^N/A: not applicable.

^g^*P*<.05.

#### Intervention Effects on Secondary and Other Health Outcomes

As a secondary outcome, self-reported fall incidence was examined at 3 time points (Table S5 in Multimedia Appendix 1). The group × time interaction for pre-post fall incidence was not statistically significant (Wald *χ*^2^_1_=0.66, *P*=.42; odds ratio [OR] 0.47, 95% CI 0.07-2.94). At the postintervention assessment, fall incidence was numerically lower in the intervention group than in the control group (5/14, 35.7% vs 7/11, 63.6%; OR 0.32, 95% CI 0.06-1.64). Although this between-group difference did not reach statistical significance (Fisher exact *P*=.24), the direction and magnitude of the effect favored the intervention group. As a secondary outcome, this finding should be interpreted as hypothesis-generating only.

A 1-year postintervention follow-up assessed fall incidence through community caregiver reports collected as part of routine Public Community Care service monitoring. No fall events were reported for any participant in either the intervention or control group during the 12-month follow-up period (intervention, 0/14; control, 0/14). A separate GEE model comparing baseline with the 12-month follow-up also showed no significant group × time interaction (Wald *χ*^2^_1_=0.13, *P*=.71; OR 1.39, 95% CI 0.24-8.05), although this model should be interpreted with caution because of quasi-complete separation resulting from the absence of events at follow-up. Although the absence of falls during follow-up is notable for this population with elevated fall risk, the floor effect precluded between-group comparisons, and the absence of a nonparticipating comparison group limited causal interpretation.

No significant group × time interactions were observed for the secondary outcomes (MoCA-K, *P*=.12; Figure-8 Walk, *P*=.65; 30-second Sit-to-Stand, *P*=.72; and 2-Minute Step Test, *P*=.62; Tables S3 and S4 in Multimedia Appendix 1) or the self-reported health outcomes (PSQI, *P*=.19; GDS-SF, *P*=.55; and WOMAC, *P*=.68).

#### Risk-Stratified Analysis

**Figure 4:**
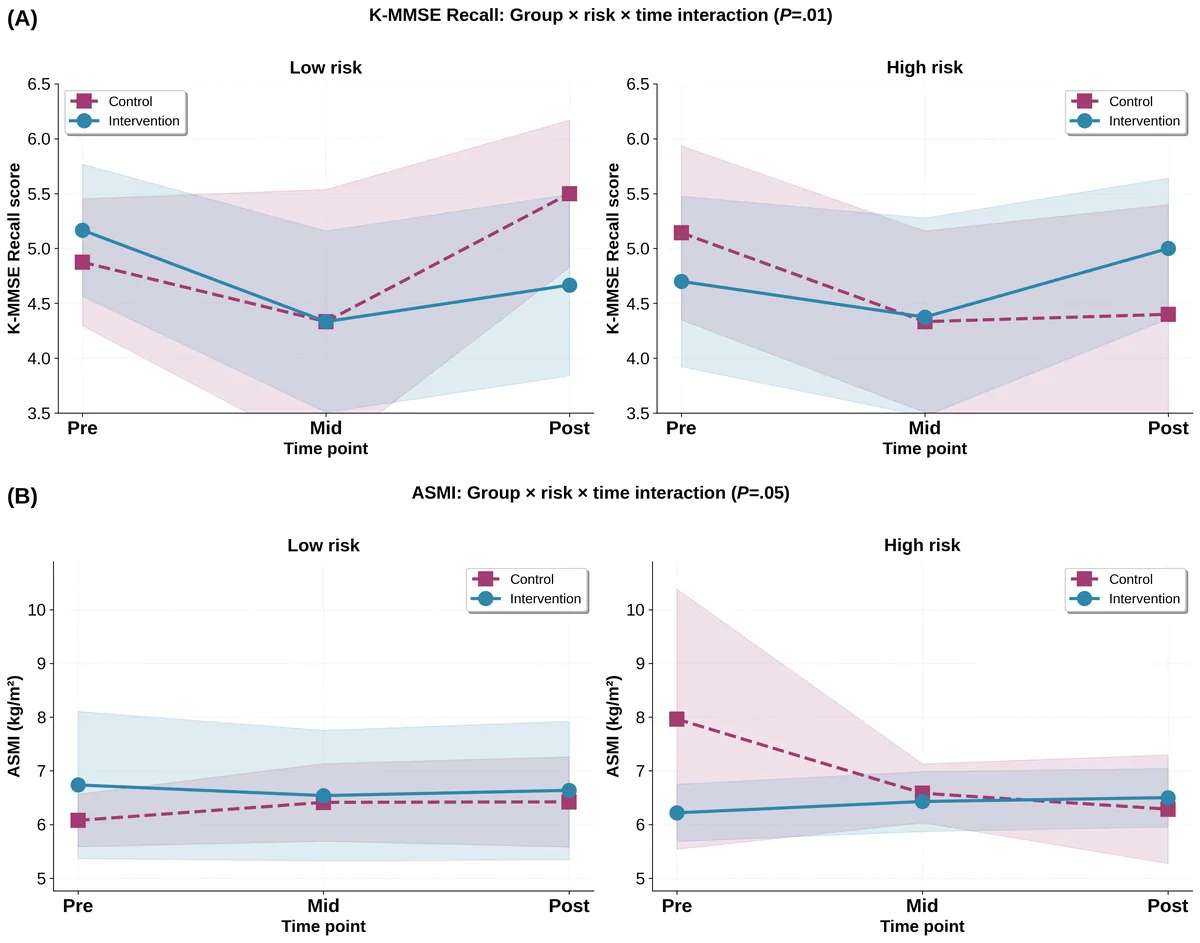
Risk-stratified intervention effects on cognitive and sensory-motor frailty outcomes. (A) Korean Mini-Mental State Examination (K-MMSE) Recall scores: a significant uncorrected group × risk × time interaction was observed (*P*=.01). In the high-risk subgroup, the intervention group showed improvement in Recall from mid- to postintervention, whereas the control group continued to decline. (B) Appendicular Skeletal Muscle Mass Index (ASMI): a group × risk × time interaction reached borderline significance (*P*=.05). In the high-risk subgroup, the intervention group maintained relatively stable muscle mass, whereas the control group showed continued decline. Lines represent estimated marginal means, and shaded areas indicate 95% CIs.

Figure 4 presents outcomes with risk-level interactions. These analyses are exploratory and hypothesis-generating because of the small subgroup cell sizes (n=5-8 per cell). High risk was defined as meeting the criteria for cognitive impairment (K-MMSE ≤21 or Montreal Cognitive Assessment [MoCA-K] <23) combined with a history of recurrent falls.

A significant uncorrected 3-way interaction (group × risk × time) was observed for K-MMSE Recall (Wald *χ*^2^_2_=6.16, *P*=.01; Figure 4A). Among the high-risk participants, the intervention group showed an improvement in Recall (4.70-5.00; *d*=0.73), whereas the control group declined (5.14-4.40). The low-risk participants showed the opposite pattern (*d*=−0.99), suggesting that cognitive effects may be concentrated among individuals with greater baseline impairment.

The 3-way interaction for ASMI reached borderline significance (Wald *χ*^2^_2_=6.00, *P*=.05; Figure 4B). Among the high-risk participants, the between-group effect size was large (*d*=0.90): the control group showed a substantial decline (8.0-6.3 kg/m^2^), whereas the intervention group maintained ASMI levels (6.2-6.5 kg/m^2^). No other outcome variables showed significant uncorrected 3-way interactions (group × risk × time), including the MoCA-K total (*P*=.71) and subscale scores (Memory, *P*=.83; Attention, *P*=.71; Language, *P*=.71; Visuospatial, *P*=.75; Orientation, *P*=.89), K-MMSE total (*P*=.71) and other subscale scores (Recall, *P*=.37; Orientation, *P*=.85; Attention, *P*=.75; Language, *P*=.71; Visuospatial, *P*=.75), and sensorimotor function measures (BBS, *P*=.82; TUG, *P*=.83; Figure-8 Walk, *P*=.75; grip strength, *P*=.81; 30-second Sit-to-Stand, *P*=.70; 2-Minute Step Test, *P*=.70; and ASM, *P*=.70).

#### Exploratory Game Performance Analyses

Post hoc exploratory correlations between game performance metrics and clinical outcomes (Figure S1 in Multimedia Appendix 1) suggested potential associations between cognitive challenge during gameplay and cognitive outcomes, although the small subgroup sizes require cautious interpretation.

## Discussion

### Principal Findings

This study examined the effects of a pressure sensor–based exergame intervention on cognitive and sensorimotor frailty in socially vulnerable older adults through 2 sequential studies. In the pilot RCT, no primary outcomes survived FDR correction, indicating that the present findings do not establish efficacy and should be interpreted as preliminary. Within this cautious framework, the pilot study (study 1), an uncontrolled single-arm design, showed pre-post improvements in balance and mobility that survived FDR correction. It also showed an uncorrected pre-post improvement in the K-MMSE Visuospatial subscale. This finding is consistent with evidence that proprioceptive training can enhance visuospatial processing through sensorimotor integration in the posterior parietal cortex [47,48]. In the RCT (study 2), nominally significant uncorrected signals were observed for ASM preservation. In exploratory risk-stratified analyses based on small subgroup cell sizes, comparable uncorrected signals emerged for K-MMSE Recall and ASMI among the high-risk participants. These exploratory patterns are hypothesis-generating only. They suggest that older adults with mild-to-moderate impairment may represent a potentially more responsive subgroup in whom the residual capacity for improvement is greater. Confirmation in adequately powered, risk-enriched trials is required.

### Comparison With Prior Work

The uncorrected preservation of ASM, together with trends toward greater grip strength, may reflect the functional loading produced by the repeated weight-shifting and stepping movements in the Brain Step program. A meta-analysis of 24 RCTs reported that exercise interventions produced a significant increase in appendicular muscle mass (+0.4 kg) among community-dwelling older adults [49], and a recent exergame RCT similarly demonstrated significant improvements in ASM following exergame-based training in residents of long-term care facilities [50]. However, these findings should be interpreted cautiously given the lack of statistical significance after FDR correction in our study.

Although the intervention group showed numerically higher BBS scores and shorter TUG times, no significant group × time interaction was observed. Participants were independently ambulatory older adults with high baseline functional levels (BBS approximately 52-53 of 56; TUG approximately 7-8 seconds). At these baseline levels, the BBS and TUG had limited capacity to detect further improvement, indicating ceiling effects. This likely reflects a mismatch between the selected outcome measures and the functional level of the sample rather than definitive evidence of no benefit. Therefore, the lack of significant effects on balance and mobility should not be interpreted as evidence of no intervention benefit because these measures may have been insufficiently sensitive to detect change in this high-functioning sample. Consistent with this interpretation, previous studies have reported limited sensitivity of the BBS for detecting subtle balance changes in high-functioning older adults [51,52] and reduced responsiveness of the TUG in individuals with preserved mobility [53]. Conventional clinical assessments such as the BBS and TUG may not adequately capture subtle changes in dynamic balance or real-world mobility, particularly under complex or dual-task conditions [54,55]. These findings underscore the need to select outcome measures that are both sensitive and ecologically valid and that are appropriately matched to the baseline functional level of the target population.

The K-MMSE Recall pattern among the high-risk participants was consistent across both studies, aligning with exergame RCTs reporting cognitive effects in individuals with MCI [19,20,56] but not in healthy [57,58] or severely impaired [59] populations. The concurrent preservation of muscle mass alongside improved recall in the high-risk participants is consistent with growing evidence that lower limb muscle function and cognitive function are bidirectionally linked [60,61] and that dual-task training engaging both motor and cognitive systems may produce benefits across both domains [12,62]. However, these concurrent patterns were observed in small, underpowered subgroups and should be regarded as hypothesis-generating signals requiring confirmation in adequately powered trials.

Self-reported fall incidence showed a nonsignificant directional reduction favoring the intervention group at the postintervention assessment (intervention, 35.7% vs control, 63.6%; *d*=−0.42). At the 12-month follow-up, no falls were reported in either group. This likely reflects the shift from retrospective self-report to prospective caregiver monitoring rather than a true treatment effect [63]. Whether proprioceptive-motor training can attenuate fall risk warrants investigation in a larger sample with longer follow-up.

### Implications for Digital Health Equity

The target population—low-income older adults living alone who were receiving Public Community Care services—is systematically underrepresented in digital health research. The successful completion of an RCT with this population, facilitated by a participatory research committee that endorsed randomization, demonstrates the feasibility of conducting rigorous evaluations within community care settings. This study completes a 3-phase research program progressing from digital health monitoring [24] through community care–mediated intervention, in which caregivers identify needs and lay workers deliver services [25], to targeted cognitive-motor training, suggesting that the standardized Public Community Care infrastructure offers a scalable platform for embedding technology-based interventions. The high retention rate despite the participants’ advanced age (mean 81.5 years), low-income status, and living alone suggests that welfare center–based delivery is a feasible model for reaching underserved populations. From a policy perspective, pressure-sensor mat systems may offer a potentially cost- and resource-efficient option for community care settings.

### Limitations

Several limitations should be considered. The small sample size (n=15 for the pilot study; n=31 for the pilot RCT) limited statistical power, and no findings survived FDR correction. The small sample size and the inclusion of outcomes across multiple domains reflected 2 design priorities: embedding the intervention within the welfare center’s existing infrastructure and adopting a multidimensional monitoring framework [24,25]. The risk-stratified subgroup analyses involved very small cell sizes (n=5-8 per subgroup), substantially limiting the reliability of subgroup effect estimates and increasing the risk of inflated type I error and unstable estimates. Although the observed effect sizes were small-to-medium overall (*d*=0.38-0.50) and medium-to-large in the high-risk subgroups (*d*=0.73-0.92), these findings should be interpreted as exploratory and hypothesis-generating and require confirmation in adequately powered trials with prespecified subgroup hypotheses. Nonetheless, low-income older adults receiving Public Community Care services constitute a hard-to-reach and underrepresented population that is rarely included in digital health research. We therefore deliberately examined the high-risk subgroup pattern that recurred from the single-arm pilot study (study 1) to the pilot RCT (study 2) and present it strictly as exploratory and hypothesis-generating to inform hypotheses, outcome selection, and sample enrichment for other investigators studying similarly constrained, hard-to-recruit populations.

Relatedly, the selected balance and mobility outcome measures were not well matched to the sample’s baseline functional level. Several balance and mobility measures, particularly the BBS and TUG, exhibited ceiling effects because participants were already highly functional at enrollment. These ceiling effects limited the sensitivity of the measures to detect change; accordingly, the null findings for balance and mobility should be interpreted as inconclusive rather than negative. Future trials should select outcome measures that are matched to the target population’s functional level and incorporate more sensitive, ecologically valid measures (eg, instrumented gait analysis or dual-task assessments).

Despite randomization, the control group had numerically higher baseline K-MMSE scores than the intervention group (25.73 vs 24.44). This minor imbalance raises the possibility of regression to the mean for cognitive outcomes. The use of a passive (no-contact) control group constitutes a key limitation of internal validity. Without an active or attention-matched comparator, any observed differences cannot be attributed unambiguously to the cognitive-motor training itself because the effects of attention, social contact, novelty, and facilitator engagement remain confounded with the intervention. Although passive control groups are often used in feasibility-stage studies, particularly when the primary objective is to evaluate delivery feasibility within an existing care infrastructure, definitive trials should employ active or sham-exergame controls [28] to strengthen causal inference.

Cognitive outcomes were limited to global screening measures (K-MMSE and MoCA-K) rather than comprehensive neuropsychological batteries, and practice effects cannot be fully excluded. Generalizability is limited by the single-site recruitment context. Multisite replication across populations with diverse socioeconomic characteristics is needed. For independently ambulatory older adults, the 1-year follow-up period may not have been sufficient to detect meaningful differences in fall incidence. The 16-week intervention period also represents a limitation for evaluating durable cognitive and sensorimotor changes.

### Conclusion

The 2 sequential studies demonstrate the initial feasibility of delivering a pressure sensor–based exergaming intervention through the existing Public Community Care infrastructure as an engaging, community-based program for socially vulnerable older adults experiencing age-related cognitive and sensorimotor decline. Designed to operate within the limited resources of a community welfare center using nonspecialist staff as facilitators, the Brain Step program offers a scalable template for multisite expansion. However, the current evidence is preliminary. The observed effects did not survive FDR correction, and the preliminary signals for muscle mass preservation and cognitive improvement were concentrated among participants with mild-to-moderate impairments, suggesting that this subgroup may benefit most from movement-mediated cognitive-motor training. Adequately powered multisite trials with longer follow-up are needed to confirm these findings and to evaluate the integration of exergame-based interventions within personalized digital health care frameworks that combine wearable-based monitoring of daily functional changes with adaptive intervention delivery.

## Appendix

Multimedia Appendix 1 Additional baseline characteristics, outcome measures, fall incidence results, and exploratory correlation analyses supporting the findings of this study.

Multimedia Appendix 2 CONSORT-eHEALTH checklist (V 1.6.1).

## Abbreviations

ASM: appendicular skeletal muscle mass
ASMI: Appendicular Skeletal Muscle Mass Index
BBS: Berg Balance Scale
CONSORT-EHEALTH: Consolidated Standards of Reporting Trials of Electronic and Mobile Health Applications and Online Telehealth
FDR: false discovery rate
GDS-SF: Geriatric Depression Scale-Short Form
GEE: generalized estimating equation
K-MMSE: Korean Mini-Mental State Examination
MoCA-K: Korean Montreal Cognitive Assessment
OR: odds ratio
PSQI: Pittsburgh Sleep Quality Index
RCT: randomized controlled trial
TUG: Timed Up and Go
WOMAC: Western Ontario and McMaster Universities Osteoarthritis Index

## Data Availability

The data contain sensitive personal and health information from socially vulnerable older adults. In accordance with the conditions of the Institutional Review Board approval (approval number KUIRB-2023-0360) and participant informed consent, the dataset cannot be made publicly available or shared with researchers who are not authorized under the original study protocol.
