# Correction: Feasibility and Preliminary Effects of a Pressure Sensor–Based Exergame on Cognitive and Sensorimotor Frailty in Low-Income Older Adults Receiving Public Community Care Services: Pilot Randomized Controlled Trial

**DOI:** 10.2196/109510

**Published:** 2026-09-09

**Authors:** Sunmi Song, Suk-Chan Hahm, Seyun Chang, Jinseung Lee, Heewon Kang, Sanghun Lee, Junesun Kim

**Affiliations:** 1 Rehabilitation Science Program, Department of Health Science, Graduate School Korea University Seoul, Seoul Republic of Korea; 2 Transdisciplinary Major in Learning Health Systems, Department of Healthcare Sciences, Graduate School Korea University Seoul, Seoul Republic of Korea; 3 Graduate School of Integrative Medicine and Health Cha University Pocheon, Gyeonggi-do Republic of Korea; 4 Geumcheon-gu, 205 Gasan digital 1-ro MIDAS Health & Technology Co. Seoul, Seoul Republic of Korea; 5 Department of Public Health Administration Daejin University Pocheon, Gyeonggi-do Republic of Korea; 6 Department of Physical Therapy/Health and Environmental Science, Undergraduate School College of Health Science Korea University Seoul, Seoul Republic of Korea

In “Feasibility and Preliminary Effects of a Pressure Sensor–Based Exergame on Cognitive and Sensorimotor Frailty in Low-Income Older Adults Receiving Public Community Care Services: Pilot Randomized Controlled Trial” [1], the authors wish to make the following correction.

The Funding section has been corrected from the following:

This study was supported by the ANCHOR through the Seoul ANCHOR Center, funded by the Ministry of Education (MOE) and the Seoul Metropolitan Government (2026-ANCHOR-01-003-01), the Basic Research Lab Program (BRL) of the National Research Foundation of Korea (NRF) (RS-2025-02303227), and the Basic Science Research Program through the National Research Foundation of Korea (NRF), funded by the Ministry of Science and ICT (MSIT) (grant number RS-2024-00348012).

The Funding section now reads:

This study was supported by the ANCHOR through the Seoul ANCHOR Center, funded by the Ministry of Education (MOE) and the Seoul Metropolitan Government (2026-ANCHOR-01-003-01), the Pioneer Research Center Program (RS-2022-NR067566) through the National Research Foundation of Korea, and the Basic Science Research Program through the National Research Foundation of Korea (NRF), funded by the Ministry of Science and ICT (MSIT) (RS-2024-00348012).

The correction will appear in the online version of the paper on the JMIR Publications website, together with the publication of this correction notice. Because this was made after submission to PubMed, PubMed Central, and other full-text repositories, the corrected article has also been resubmitted to those repositories.
