# Supplementary material for: Feasibility and Preliminary Effects of a Pressure Sensor–Based Exergame on Cognitive and Sensorimotor Frailty in Low-Income Older Adults Receiving Public Community Care Services: Pilot Randomized Controlled Trial
Source: JMIR Aging. 2026 Jul 24;9:e93467. doi: 10.2196/93467 (PMC13451870; doi:10.2196/93467)
Supplement: Supplementary file 2 [file aging_v9i1e93467_app1.docx]

**Supplemental Table S1.** **Baseline characteristics of the pilot test participants (n = 15)**

| **Variable** | **Mean (SD) or N (%)** |
| --- | --- |
| **Demographic variables** |  |
| Age (years) | 80.20 (6.88) |
| Sex (Women, n (%)) | 12 (80%) |
| Years of education | 9.11 (4.73) |
| Working, Yes | 5 (33.3%) |
| **Monthly income (KRW), n (%)** | |
| Less than 500,000 | 3 (20%) |
| 500,001-1,000,000 | 5 (33.3%) |
| 1,000,000 or more | 7 (46.7%) |
| **Charlson Comorbidity Index (CCI), n (%)** |  |
| 0 | 11 (73.3%) |
| 1-2 | 4 (26.7%) |
| 3 or more | 0 (0%) |
| **Cognitive functions** | |
| MMSE, total | 23 (3.13) |
| **Physical Functions** | |
| BMI (kg/m²), mean (SD) | 25.26 (3.70) |
| Berg Balance Scale | 51.10 (3.92) |
| Appendicular Skeletal Muscle (kg) | 14.65 (2.18) |
| Appendicular Skeletal Muscle Index (kg/m²) | 6.14 (0.74) |
| Grip Strength (kg) | 21.12 (4.36) |
| **Other health outcomes** | |
| PSQI | 6.86 (3.42) |
| GDS | 2.5 (2.72) |
| WOMAC Total | 13.80 (7.33) |

**Note.** US $1=1344 Korean won (KRW). CCI = Charlson Comorbidity Index; MMSE = Mini-Mental State Examination; BMI = Body Mass Index; WOMAC: Western Ontario and McMasters Universities

**Supplemental** **Table S2. Comparison of Cognitive and Sensory-motor Function Over Time for Pilot Testing**

| **Variable** | **Intervention Group (n = 15)** | | **p** | **FDR**  **p** |
| --- | --- | --- | --- | --- |
|  | **Pre** | **Post** |  |  |
| **Primary Outcomes** |  |  |  |  |
| **K-MMSE †** |  |  |  |  |
| Total | 24.47 (3.23) | 24.53 (2.85) | 0.835 | .835 |
| Orientation | 9.47 (0.99) | 9.40 (1.12) | 0.738 | — |
| Recall | 5.47 (0.99) | 5.47 (0.64) | 1.000 | — |
| Attention | 4.07 (1.44) | 4.00 (1.41) | 0.653 | — |
| Language | 7.87 (0.35) | 7.80 (0.41) | 0.301 | — |
| Visuospatial | 0.60 (0.51) | 0.87 (0.35) | 0.020* | — |
| **Berg Balance Scale (score)** | 51.10 (3.92) | 53.20 (2.70) | <.001* | .002* |
| **Timed Up and Go (sec)** | 8.45 (2.47) | 7.41 (2.27) | <.001* | .002* |
| **ASM (kg)** | 14.65 (2.18) | 14.31 (2.44) | 0.041* | .071 |
| **ASMI (kg/m²)** | 6.14 (0.74) | 5.99 (0.74) | 0.047* | .071 |
| **Grip Strength (kg)** | 22.01 (4.58) | 22.95 (5.36) | 0.095 | .114 |
| **Secondary Outcomes** |  |  |  |  |
| **Figure-8 Walk (sec)** | 35.83 (9.96) | 32.44 (10.36) | 0.002* | .006* |
| **30-second Sit-to-Stand (reps)** | 16.20 (5.85) | 19.73 (6.05) | 0.007* | .011* |
| **2-minute Step Test (steps)** | 90.73 (23.08) | 98.27 (19.73) | 0.017* | .017* |

Note. Values are presented as Mean (SD). Pre-post changes were analyzed using Generalized Estimating Equations (GEE) with an exchangeable correlation structure. Benjamini-Hochberg FDR correction (q = 0.05) was applied within the six IRB-specified primary outcomes (K-MMSE total, BBS, TUG, ASM, ASMI, grip strength). Secondary outcomes reported with unadjusted p-values. K-MMSE = Korean Mini-Mental State Examination; ASM = appendicular skeletal muscle mass; ASMI = appendicular skeletal muscle mass index (ASM/height²); † Analyses with K-MMSE outcomes were adjusted for years of education. * p < 0.05

Osteoarthritis Index; GDS = Geriatric Depression Scale; PSQI = Pittsburg Sleep Quality Index.

# **Supplemental Table S3. Cognitive frailty outcomes with mid-intervention data (n = 31)**

|  | **Intervention (n=16)** | | | **Control (n=15)** | | | **Group × Time** | | **d** |
| --- | --- | --- | --- | --- | --- | --- | --- | --- | --- |
| **Variable** | **Pre** | **Mid** | **Post** | **Pre** | **Mid** | **Post** | **p** | **FDR p** |  |
| **Primary Outcomes** | | | | | | | | | |
| **K-MMSE** | | | | | | | | | |
| Total | 24.44 (2.90) | 21.14 (2.63) | 22.00 (3.01) | 25.73 (1.87) | 22.25 (2.38) | 23.27 (2.24) | .275 | .382 | -0.17 |
| Orientation | 9.12 (0.89) | 9.43 (0.65) | 9.36 (1.08) | 9.13 (1.30) | 9.33 (0.98) | 9.73 (0.47) | .362 | — | -0.63 |
| Recall | 4.88 (1.09) | 4.36 (1.15) | 4.86 (0.95) | 5.00 (0.93) | 4.33 (1.23) | 5.00 (1.10) | .089 | — | -0.29 |
| Attention | 2.38 (1.75) | 2.07 (1.44) | 2.64 (1.69) | 3.13 (1.36) | 3.08 (1.31) | 3.00 (1.55) | .366 | — | 0.30 |
| Language | 7.56 (0.51) | 7.57 (0.65) | 7.43 (0.76) | 7.60 (0.63) | 7.67 (0.49) | 7.73 (0.47) | .271 | — | -0.08 |
| Visuospatial | 0.50 (0.52) | 0.71 (0.47) | 0.71 (0.47) | 0.87 (0.35) | 0.83 (0.39) | 0.82 (0.40) | .121 | — | 0.69 |
| **Secondary Outcomes** | | | | | | | | | |
| **MoCA-K** | | | | | | | | | |
| Total | 18.81 (4.18) | 20.64 (3.15) | 20.50 (3.67) | 18.93 (4.08) | 19.50 (3.00) | 21.18 (3.46) | .117 | .468 | -0.42 |
| Memory | 2.19 (1.76) | 1.71 (1.27) | 2.07 (1.49) | 1.53 (1.51) | 0.83 (1.27) | 1.73 (1.42) | .700 | — | -0.47 |
| Attention | 3.25 (1.98) | 3.50 (1.22) | 3.14 (1.51) | 3.60 (1.59) | 3.75 (0.97) | 3.55 (0.82) | .215 | — | -0.27 |
| Language | 4.44 (1.26) | 4.79 (1.42) | 4.64 (1.01) | 4.27 (1.33) | 4.67 (0.98) | 4.55 (1.51) | .499 | — | 0.22 |
| Visuospatial | 2.06 (1.18) | 2.71 (0.83) | 2.93 (1.21) | 2.27 (1.03) | 2.42 (1.08) | 2.82 (0.75) | .205 | — | 0.30 |
| Orientation | 5.12 (0.81) | 5.50 (0.65) | 5.36 (1.08) | 5.20 (1.15) | 5.83 (0.39) | 5.73 (0.47) | .456 | — | -0.54 |

**Note.** Values are mean (SD). Mid = 8-week assessment. Group × Time interaction effects analyzed using GEE with exchangeable correlation structure. Cohen’s d calculated from between-group differences in change scores divided by pooled SD. Benjamini-Hochberg FDR correction (q = 0.05) applied within the six IRB-specified primary outcomes (K-MMSE total, Berg Balance Scale, Timed Up and Go , ASM, ASMI, grip strength; 6 comparisons). Secondary outcomes (MoCA-K total, Figure-8 Walk, 30-sec Sit-to-Stand, 2-Min Step Test) reported with unadjusted p-values. * p < .05 (uncorrected). No outcomes reached significance after FDR correction. K-MMSE = Korean Mini-Mental State Examination; MoCA-K = Montreal Cognitive Assessment-Korean; ASM = appendicular skeletal muscle mass; ASMI = appendicular skeletal muscle mass index (ASM/height²). No outcomes reached significance after FDR correction.

# **Supplemental Table S4. Sensory-motor frailty outcomes with mid-intervention data (n = 31)**

|  | **Intervention (n=16)** | | | **Control (n=15)** | | | **Group × Time** | | **d** |
| --- | --- | --- | --- | --- | --- | --- | --- | --- | --- |
| **Variable** | **Pre** | **Mid** | **Post** | **Pre** | **Mid** | **Post** | **p** | **FDR p** |  |
| **Primary Outcomes** | | | | | | | | | |
| Berg Balance Scale | 52.88 (1.76) | 54.11 (1.38) | 53.82 (1.15) | 52.73 (2.46) | 53.75 (1.92) | 53.82 (2.21) | .054 | .108 | -0.27 |
| Timed Up and Go (sec) | 7.67 (1.80) | 7.42 (0.91) | 6.86 (0.82) | 7.97 (1.99) | 8.03 (1.22) | 7.54 (1.34) | .650 | .650 | -0.03 |
| ASM (kg) | 15.35 (4.48) | 15.58 (4.44) | 15.77 (4.56) | 17.03 (6.00) | 15.87 (2.48) | 15.48 (3.06) | .039* | .108 | 0.39 |
| ASMI (kg/m²) | 6.41 (1.22) | 6.48 (1.11) | 6.56 (1.16) | 6.96 (2.40) | 6.50 (0.77) | 6.36 (1.04) | .052 | .108 | 0.38 |
| Grip Strength (kg) | 21.16 (6.88) | 24.46 (5.42) | 23.27 (5.37) | 20.79 (4.61) | 21.43 (2.24) | 19.71 (3.73) | .318 | .382 | 0.50 |
| **Secondary Outcomes** | | | | | | | | | |
| Figure-8 Walk (sec) | 31.59 (5.71) | 31.67 (4.49) | 29.04 (3.67) | 35.09 (7.81) | 33.67 (5.66) | 32.84 (6.29) | .655 | .721 | 0.15 |
| 30-sec Sit-to-Stand | 14.75 (6.05) | 18.93 (4.07) | 16.79 (3.83) | 11.93 (3.81) | 16.33 (4.23) | 15.09 (3.39) | .721 | .721 | -0.43 |
| 2-min Step Test | 90.88 (16.49) | 93.07 (18.27) | 81.86 (22.46) | 84.80 (14.97) | 89.83 (11.33) | 78.18 (13.84) | .619 | .721 | -0.39 |

**Note.** Values are mean (SD). Group × Time interaction effects analyzed using GEE with exchangeable correlation structure. Cohen’s d calculated from between-group differences in change scores divided by pooled SD. Benjamini-Hochberg FDR correction (q = 0.05) applied within the six IRB-specified primary outcomes (K-MMSE total, BBS, TUG, ASM, ASMI, grip strength). Secondary outcomes reported with unadjusted p-values. * p < .05 (uncorrected). No outcomes reached significance after FDR correction. ASM = appendicular skeletal muscle mass; ASMI = appendicular skeletal muscle mass index (ASM/height²). No outcomes reached significance after FDR correction.

**Supplemental Table S5. Self-Reported Fall Incidence by Group Across Three Time Points**

| **Time point** | **Intervention,**  **n/N (%)** | **Control,**  **n/N (%)** | **Group × Time interaction p** | **Cohen's d** |
| --- | --- | --- | --- | --- |
| Pretest (baseline) | 4/16 (25) | 5/15 (33.3) | - | — |
| Post-intervention (16 weeks) | 5/14 (35.7) | 7/11 (63.6) | .418 | −0.42 |
| 12-month follow-up | 0/14 (0.0) | 0/14 (0.0) | .714 | 0.18 |

**Note. Fall incidence was assessed as a secondary outcome through self-report at baseline (past 12 months), at immediate post-intervention (past 16 weeks), and at 12-month follow-up (through community caregiver reports). Group × Time interaction p-values were obtained from generalized estimating equation (GEE) models with the participant as the cluster, an exchangeable working correlation, and robust (sandwich) standard errors; the binomial family with a logit link was used. Cohen's d was approximated from the log-odds ratio using d = ln(OR) × √3/π (Chinn, 2000); a negative value indicates a smaller increase in fall incidence in the intervention group compared with the control group. Interpretation of the 12-month follow-up model should be made with caution due to quasi-complete separation (zero events in both groups at follow-up).**

(A)

(B)

**Supplemental Figure S1. Exploratory correlations between game performance indicators and clinical outcome changes among intervention participants.** **(A) K-MMSE Recall correlations in the high-risk subgroup (n=8), showing associations with Maze Game, Side Reach, Antarctic Adventure, and Cognitive Training metrics. (B) ASMI correlations in the high-risk subgroup (n=8), showing associations with Side Reach, Maze Game, and Antarctic Adventure metrics. Point estimates (diamonds) represent Pearson correlation coefficients, with horizontal lines indicating 95% confidence intervals computed via Fisher's z-transformation.**

Blue indicates positive and orange indicates negative correlations for pre-to-post changes; green indicates positive and purple indicates negative correlations for mid-to-post changes. These correlations are exploratory and should be interpreted with caution given the small subsample sizes. *p<0.05, **p<0.01.

**Supplemental Methods**

**Detailed Brain Step Game Descriptions**

The Brain Step cognitive and sensory-motor function training system (Figure 1D) comprised seven exergames that combined proprioceptive-motor movement with progressive cognitive demands. The exergame designs were inspired by classic arcade games from the 1980s–1990s to enhance engagement through nostalgic familiarity. The games included:

(1) Antarctic Expedition—shifting weight between toes and heels to navigate a penguin around obstacles, targeting anteroposterior weight shift control.

(2) Amazon Balance Game—lateral weight shifting for spatial orientation training, targeting mediolateral balance control.

(3) Maze Navigation—shifting body weight to navigate through on-screen mazes, combining spatial planning with continuous weight transfer.

(4) Stepping Stones—stepping on designated areas in response to visual cues to train balance and reaction time.

(5) Rhythm Game—rhythmic foot movements following directional cues, integrating temporal processing with motor coordination.

(6) Mole Catching—quickly stepping on targets appearing at random positions, training rapid weight shifting and visuomotor response.

(7) Multi-domain Cognitive Training—quizzes targeting orientation, memory, language, visuospatial cognition, and arithmetic, with participants responding by stepping on symbol-coded answer zones.

**Fall Incidence Statistical Analysis Details**

For the secondary analysis of fall incidence, between-group differences at each time point were examined using Fisher’s exact test (two-sided) given the binary outcome and modest sample size. Effect sizes were summarized as risk differences with 95% confidence intervals computed using the Newcombe hybrid score method, together with odds ratios and their 95% Wald confidence intervals on the log scale. Generalized estimating equation (GEE) models with binomial distribution and logit link, including main effects of group and time and their interaction, were also fitted to exploit the longitudinal structure (one model for pretest-to-posttest and a separate model for pretest-to-12-month follow-up). Cohen’s d was approximated from the log-odds ratio using d = ln(OR) × √3/π (Chinn, 2000). Results should be interpreted with caution given the small sample size.
